# The Proteomic Landscape of the Coronary Accessible Heart Cell Surfaceome

**DOI:** 10.1002/pmic.202400320

**Published:** 2025-01-10

**Authors:** Iasmin Inocencio, Alin Rai, Daniel Donner, David W. Greening

**Affiliations:** ^1^ Baker Heart and Diabetes Institute Melbourne Victoria Australia; ^2^ Baker Department of Cardiovascular Research Translation and Implementation La Trobe University Melbourne Victoria Australia; ^3^ Baker Department of Cardiometabolic Health University of Melbourne Melbourne Victoria Australia; ^4^ Central Clinical School Monash University Melbourne Victoria Australia

**Keywords:** cell surface, heart, mass spectrometry, proteomics, surfaceome

## Abstract

Cell surface proteins (surfaceome) represent key signalling and interaction molecules for therapeutic targeting, biomarker profiling and cellular phenotyping in physiological and pathological states. Here, we employed coronary artery perfusion with membrane‐impermeant biotin to label and capture the surface‐accessible proteome in the neo‐native (intact) heart. Using quantitative proteomics, we identified 701 heart cell surfaceome accessible by the coronary artery, including receptors, cell surface enzymes, adhesion and junctional molecules. This surfaceome comprises to 216 cardiac cell‐specific surface proteins, including 29 proteins reported in cardiomyocytes (CXADR, CACNA1C), 12 in cardiac fibroblasts (ITGA8, COL3A1) and 63 in multiple cardiac cell types (ICAM1, SLC3A2, CDH2). Further, this surfaceome comprises to 53 proteins enriched in heart tissue compared to other tissues in humans and implicated in cardiac cell signalling networks involving cardiomyopathy (CDH2, DTNA, PTKP2, SNTA1, CAM, K2D/B), cardiac muscle contraction and development (ENG, SNTA1, SGCG, MYPN), calcium ion binding (SGCA, MASP1, THBS4, FBLN2, GSN) and cell metabolism (SDHA, NUDFS1, GYS1, ACO2, IDH2). This method offers a powerful tool for dissecting the molecular landscape of the coronary artery accessible heart cell surfaceome, its role in maintaining cardiac and vascular function, and potential molecular leads for studying cardiac cell interactions and systemic delivery to the neo‐native heart.

**Abbreviations**
CM: cardiomyocyte
CVD: cardiovascular disease
DTT: dithiothreitol
EC: endothelial cell
ECM: extracellular matrix
FB: fibroblast
GO: Gene Ontology
GPI: glycosylphosphatidylinositol
IAA: indoleacetic acid
OCT: optimal cutting temperature
PBS: phosphate buffered saline
RTK: receptor tyrosine kinase
SMC: smooth muscle cell

## 1 Introduction

The heart surfaceome, comprising cell surface proteins [1, 2], mediates interactions between cardiac cells and their microenvironment. These interactions are vital for maintaining organ homeostasis, which encompasses synchronised mechanical contraction, transport of metabolites, and integrity of the structural and cellular matrix architecture [3]. Dysregulation of the surfaceome is implicated in various cardiovascular diseases (CVDs) [2], including cardiomyopathy, atrial fibrillation, pathological hypertrophy, myocardial infarction, reperfusion injury and heart failure [4, 5, 6]. Functionally, the surfaceome includes receptors, transporters, enzymes, cell adhesion molecules and other membrane‐embedded or tethered proteins with an extracellular spanning sequence. Thus, the cardiac cell surfaceome represents an important class of molecules for therapeutic targeting [7] and CVD management. Importantly, a portion of the cardiac cell surfaceome remains accessible to the circulation [8, 9, 10].

This surfaceome subset is important for the recruitment of innate immune cells to injury sites in the heart, crucial for the body's response to pathogen‐driven and sterile inflammatory processes, such as those seen in reperfusion injury [11], which can significantly impact the disease's progression and outcome [12, 13]. Importantly, these proteins are also being investigated as potential targeting moieties for cardiac [14] and selective organ delivery [15, 16, 17, 18, 19, 20, 21]. However, a detailed analysis of the coronary‐accessible cardiac surfaceome of the intact (neo‐native) heart remains unknown.

Defining the surfaceome using quantitative mass spectrometry (MS) remains challenging due to the intrinsic properties of cell surface proteins, such as low abundance, post‐translational modifications and/or hydrophobic regions that make them difficult to identify [22, 23, 24]. Further, current approaches to defining the heart surfaceome have employed the disruption of cardiac tissue and cell isolation and sorting before surface labelling [2, 25]. These approaches do not capture the neo‐native state of the heart surfaceome [26], and likely introduce alterations in membrane proteome [27, 28, 29].

Here, by retaining the neo‐native state of the heart, we combine a protein‐centric workflow that enables the labelling, capture and detection of the murine heart surfaceome landscape. We employed aortic cannulation combined with biotin and ultra‐high‐sensitivity MS‐based proteome analysis to define the cardiac cell surfaceome. Here, we employed Sulfo‐NHS‐SS‐Biotin that enables selective reactivity and covalent attachment to primary amines on surface‐expressed proteins, as previously applied to cell‐based labelling [30] and cell surface peptide enrichment strategies [31, 32]. We further highlight proteome heterogeneity of the cardiac surfaceome, and surface marker proteins of the heart accessible to the circulation. This current study provides a protein‐centric resource for cardiac cell surfaceome, and potential molecular leads for interaction and systemic delivery to the murine neo‐native heart.

## 2 Result

### 2.1 Workflow for Capturing the Surface Accessible Heart Proteome Through the Coronary Artery

**FIGURE 1:**
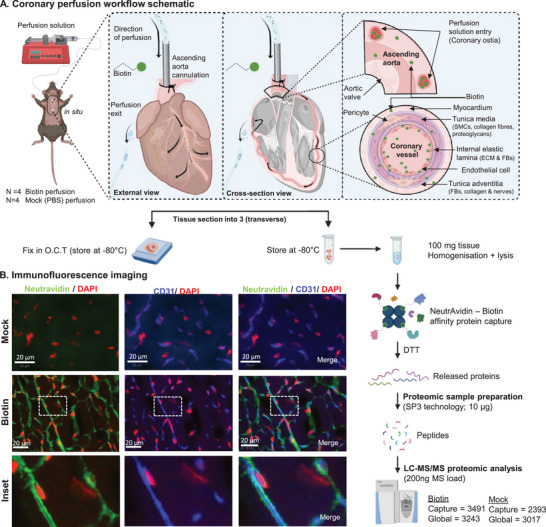
Workflow for capturing the surface accessible heart proteome through the coronary artery. (A) Schematic of in situ coronary perfusion of mouse hearts (Biotin, *N* = 4) with biotin saline solution and phosphate buffered saline (PBS) (Mock, *N* = 4), and downstream tissue analysis. Following rapid ice‐cold perfusion (PBS wash; 2 min, biotin solution; 4 min) hearts were excised and processed for either fluorescence microscopy analysis or proteomic profiling. (B) Fluorescence microscopy imaging of cardiac (murine) tissue to highlight coronary vasculature using CD31 (blue) and biotinylated proteins using FITC‐conjugated Neutravidin (green). Nuclei stained using DAPI (red), *N* = 4, representative image is shown.

To capture proteins in hearts that are accessible via the coronary vasculature, we employed a coronary biotin perfusion workflow combined with proteome profiling (Figure 1A). Here, we have employed a modified terminal rodent perfusion method [33]. Briefly during perfusion, the needle was oriented into the ascending aorta for in‐situ terminal coronary vasculature perfusion. We initially perfused wash solution (ice‐cold phosphate buffered saline [PBS], 2 min) to remove blood components. We then perfused ice‐cold membrane impermeant biotin solution (Biotin) or PBS (Mock) (2 min), which enter the cardiac tissue via the heart's right and left coronary arteries [34, 35] (Figure 1A). This ester derivative of biotin (Sulfo‐NHS‐SS‐Biotin) reacts with the amine group of proteins to label the cell surface protein network [23, 30, 36]. Moreover, this biotin derivative was selected for its aqueous solubility, limited diffusion through biological membranes and cleavable disulfide linker [37]. Although prior studies have employed ex vivo perfusion and tissue (kidney) biotinylation [38], these studies have reported associated intracellular protein labelling/detection. The final perfusion consisted of ice‐cold Tris buffered saline (4 min) to remove unbound biotin. We then analysed the heart using immunofluorescence and proteome profiling. Confocal microscopy revealed that compared to Mock perfusion, Biotin perfused heart tissues contained an abundance of biotin signal colocalised with endothelial cell (EC) surface protein CD31 (Figure 1B, Figure S1). The inset images show no obvious leaching of the biotin perfusion into the parenchymal regions of the mouse heart, restricted within the coronary vessels.

Next, we captured the biotinylated proteins using Neutravidin beads. Captured proteins were eluted from beads using dithiothreitol (DTT) and subjected to untargeted nLC‐MS/MS analysis. A stringent identification criterion of 1% false discovery rate (FDR) for peptide/protein was employed. For proteins that were identified in at least 3/4 proteome dataset, we identified 3491 and 2393 proteins in Biotin and Mock samples, respectively (Table S1). In contrast, we identified 3243 and 3017 proteins, respectively, in the global proteomes for Biotin and Mock samples (Table S1). We further performed a validation biotin‐capture dataset, using *N* = 3 independent hearts for this subsequent analysis (Table S15).

### 2.2 Defining the Surface Accessible Heart Proteome Landscape

**FIGURE 2:**
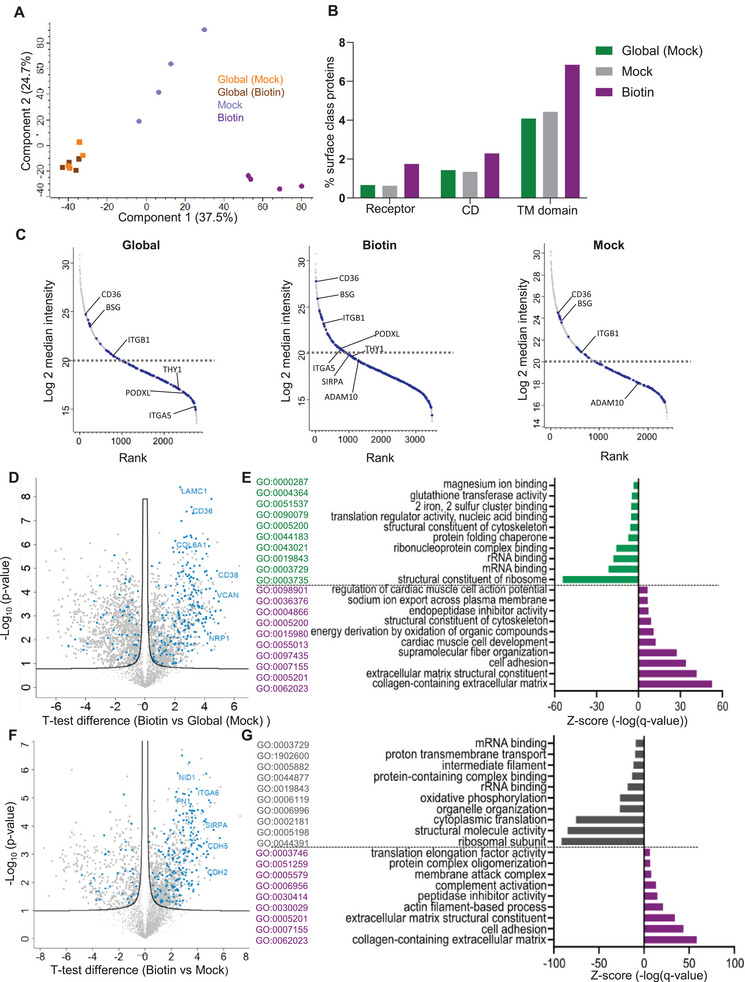
Defining the surface accessible heart proteome landscape. (A) Principal component analysis of Global (Mock), Global (Biotin), Biotin and Mock proteomes. (B) The proportion of identified surface protein categories in each proteome as listed in the cell‐surface protein atlas (CSPA) and SURFY databases. (C) Waterfall plots of surface protein abundance of Global (Mock), Biotin and Mock proteomes. (D) Volcano plot highlights differentially abundant proteins in Biotin versus global (Mock) proteomes. Highlighted proteins (blue) are annotated in the CSPA database as surface proteins. (E) Gene Ontology functional enrichment terms of the top 10 most significant (false discovery rate [FDR] < 0.05) pathways enriched in the Biotin versus Global (Mock) proteomes. (F) Volcano plot depicting differentially abundant proteins in Biotin versus Mock proteomes. Highlighted proteins (blue) are annotated in the CSPA database as surface proteins. (E) Gene Ontology functional enrichment terms of the top 10 most significant (FDR < 0.05) pathways enriched in the Biotin versus Mock proteomes.

Principal component analysis revealed that while global proteomes (Biotin vs. Mock) were indistinguishable, Biotin‐captured proteomes were distinct from Mock and global proteomes (Figure 2A). To ascertain whether the Biotin‐captured proteome was enriched in classical cell surface proteins, we mapped experimentally verified cell surface protein atlas (CSPA) [39] proteins and cell surfaceome predictor SURFY [1] proteins to our global, control capture and captured proteome profiles (Table S2). Our data show that a higher proportion of surface annotated proteins were represented in the Biotin proteome dataset (175 proteins, 41 unique) (Table S1). These proteins include receptors (including receptor tyrosine kinases [RTKs]), cell surface enzymes (e.g., proteases), bonified cell membrane cluster of differentiation components, and various transmembrane (TM) proteins including adhesion and junctional molecules (cadherins, catenin), integrins, ephrins, plexins and sodium/calcium exchanger proteins (Figure 2B, Table S2). These proteins also include glycosylphosphatidylinositol (GPI)‐anchored proteins (16 in Biotin vs. 8 in Global) that lack TM domains.

More importantly, these surface proteins displayed a higher abundance in Biotin capture compared to Mock or the global proteome (Figure 2C, Table S3). These enriched surface components included various integrins, and surface proteins CD36 and BSG increased in intensity following Biotin capture. Further, we report surface proteins uniquely identified in the Biotin data including TEK, ADAM 17, VCAM1, JAM3, ITGB5, EPHB3 and PLXNA1 (Table S1).

We next performed pair‐wise analysis to identify differentially abundant proteins in Biotin versus global proteome datasets. For this, only proteins identified in at least 3/4 proteomes in one dataset were considered. We identified 1092 proteins significantly enriched (*q* < 0.05) in Biotin vs. global (Mock) proteome (Figure 2D, Table S4). Of these, 224/1092 proteins in Biotin were CSPA surface annotated proteins (blue highlight). For transparency, we have further reported non‐surface annotated proteins (based on CSPA/SURFY) in our Biotin capture (12.5% of the captured proteome) that also include surface proteins (54 proteins, e.g., Clic4, Tjp1 and Ly6c1) as identified in UniProt based on subcellular location (Table S16).

Gene Ontology (GO, biological process) enrichment analysis revealed various networks associated with extracellular matrix (ECM), cell and substrate adhesion and cardiac muscle cell development (Figure 2E, Table S6). In contrast, RNA binding proteins and cytoskeletal proteins, typical of intracellular proteins were abundant in global proteome (Figure 2E, Table S7). These data strongly suggest successful enrichment of cell surface proteins in this study.

Moreover, 998 proteins were enriched in Biotin versus Mock proteomes. These categories and protein networks were further enriched in comparison between Biotin and Mock, where we identified 206/998 proteins in Biotin were CSPA annotated as surface‐associated (Figure 2F, Table S5). Similarly, surface proteins were enriched in cell adhesion, collagen‐containing ECM, actin filament and membrane attack components (Figure 2G, Table S8). The Mock proteome exhibited proteins associated with gene expression and energy‐dependent transport processes, such as nucleosomal DNA binding and GDP binding, molecular functions not typically observed in surfaceome activity (Figure 2G, Table S9).

### 2.3 Capturing the Cardiac and Endothelial Surfaceome

**FIGURE 3:**
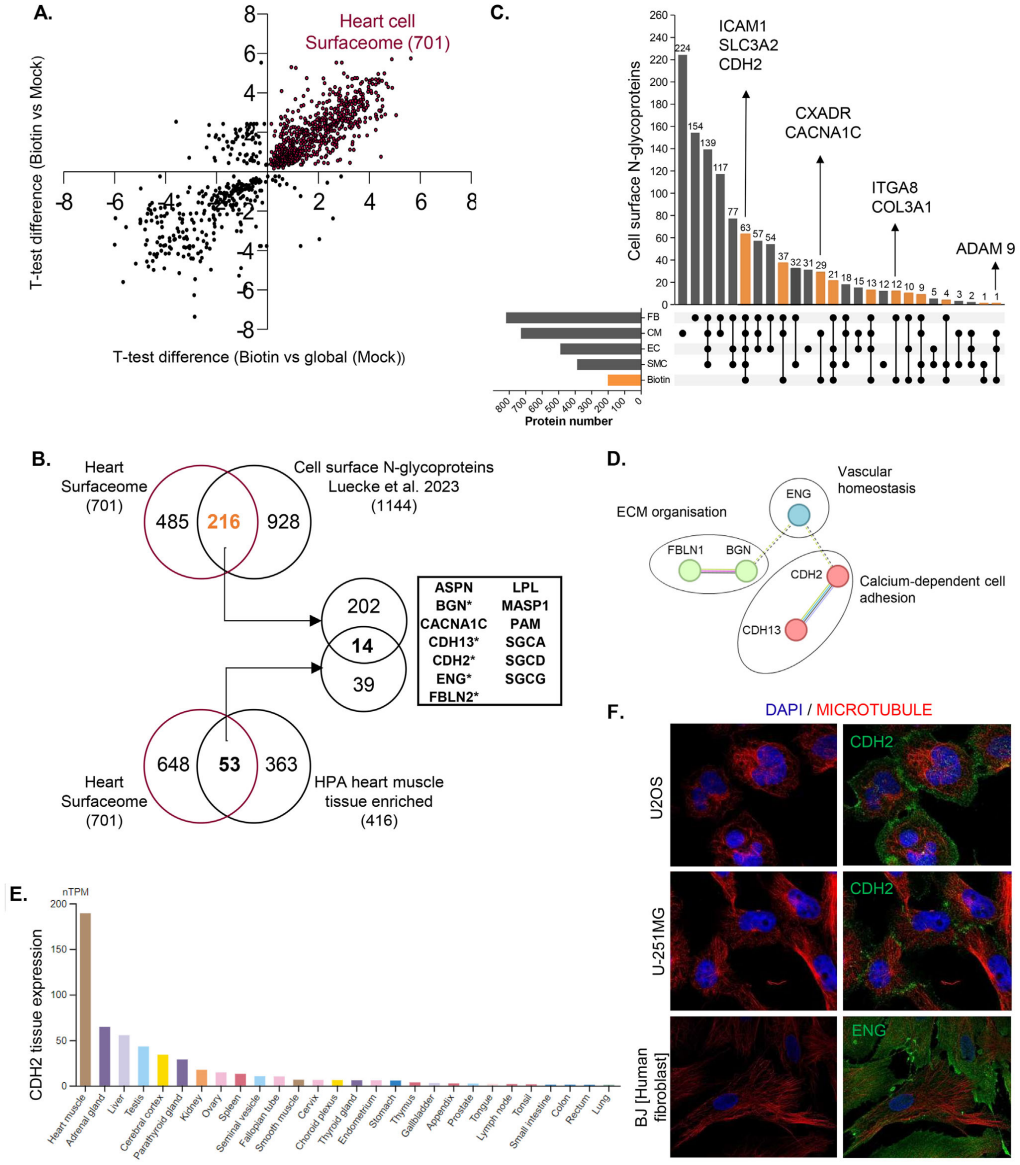
The cardiac surfaceome encompass cardiac cell heterogeneity. (A) Scatter plot illustrating the relationship between significantly enriched Biotin proteomes (*t* test differences ≥ 0.05) compared to global (Mock) and Mock proteomes. (B) Venn diagram highlighting heart cell surfaceome enriched in either (i) human cardiac cell surfaceome [2] (Luecke et al. 2023), (ii) heart enriched tissue expression from Human Protein Atlas (HPA) [40], or both (14 proteins). * Indicates proteins reported as markers for cardiac endothelial cells [2]. (C) UpSet plot visualising cardiac surfaceome distribution in human cardiac cell types (cardiomyocytes [CMs], cardiac fibroblasts [CFs], cardiac microvascular endothelial cells [ECs] and coronary artery smooth muscle cells [SMCs] [2]). (D) Network visualisation by Cytoscape and STRING of biological processes of heart EC protein markers. (E) Bar plot of CDH2 transcript expression (nTPM; transcripts per million) in different human tissues, HPA [40]. (F) Fluorescence microscopy of CDH2 (green) in human cell lines U2OS and U‐251MG and ENG (green) in BJ [human fibroblast] cells. Microtubules stained in red, nuclei in blue. Images obtained from HPA, subcellular [44].

We next focused on proteins that were significantly enriched in both Biotin versus Mock (*q* < 0.05) as well as Biotin versus Global (*q* < 0.05). This resulted in 701 proteins which represent high‐confidence cell surface‐accessible proteins in the heart (Figure 3A, Table S10). We validated in an independent heart set (*N* = 3) using the same biotin label/capture and proteome strategy to reveal 640/701 proteins coidentified (91%) in the heart cell surfaceome (Figure S4A, Table S15). Of these 701 proteins, 216 (Figure 3B) were previously reported as cardiac cell surface proteins [2]. Further, 29 have been previously identified as cardiomyocyte (CM) surface protein, 12 as fibroblast (FB) surface proteins and 1 as a CM and EC surface protein (Table 1). Moreover, 63 were ubiquitously identified as surface proteins for CM, FB, EC and smooth muscle cell (SMC) (Figure 3C, Table S11). CM‐specific proteins include CACNA1C and CXADR. FB‐specific proteins include ITGA8 and COL3A1. Interestingly we did not find EC‐specific surface proteins; however, we identified 21 proteins that were identified in EC, SMC and FB, but not in CM. A total of 108 proteins were identified as EC surface proteins (Figure 3C, Table S11). A UniProt search for proteins annotated with ‘vascular’ and ‘cell surface’ (GO:009986) identified 19 proteins overlapping with the heart cell surfaceome. Of these, 12 were classified as components of the EC cardiac surfaceome (Table 3). Notably, five proteins (VCAM1, CD34, JAM2, CD47 and PLVAP) were identified as classical endothelial markers, consistent with their established roles in endothelial biology and vascular function.

**TABLE 1:** Highlighted cell types of the heart surfaceome.

Cardiac surfaceome cell type (PMID: 36950336)	Gene name	Protein name	Protein class	Precursor ion intensity (log2, median) Biotin (surfaceome)
Cardiomyocyte	AFM	Afamin	Transport	20.2
	ALB	Albumin	Transport	28.2
	C4BPA	C4b‐binding protein	Adaptor	19.1
	C6	Complement component C6	Adaptor	17.9
	C8B	Complement component C8 beta chain	Adaptor	17.5
	CACNA1C	Voltage‐dependent L‐type calcium channel subunit alpha‐1C	Ion channel	17.8
	CFB	Complement factor B	Adaptor	20.7
	CFI	Complement factor I	Adaptor	19.4
	CHL1	Neural cell adhesion molecule L1‐like protein	Cell adhesion	17.7
	CP	Ceruloplasmin	Oxidase	21.6
	CPB2	Carboxypeptidase B2	Enzyme	17.3
	CXADR	Coxsackievirus and adenovirus receptor homolog	Cell adhesion	19.6
	FGG	Fibrinogen gamma chain	Coagulation	23.9
	FKBP9	Fibrinogen gamma chain	Chaperone	16.8
	GPLD1	Phosphatidylinositol‐glycan‐specific phospholipase D	Enzyme	17.4
	HRG	Histidine‐rich glycoprotein	Transport/regulatory	20.4
	ITIH1	Inter‐alpha‐trypsin inhibitor heavy chain H1	Protease	21.9
	ITIH4	Inter alpha‐trypsin inhibitor, heavy chain 4	Protease	16.1
	KLKB1	Plasma kallikrein	Enzyme	18.6
	LIFR	Leukaemia inhibitory factor receptor	Receptor	18.3
	LPL	Lipoprotein lipase	Lipid enzyme	21.5
	LUM	Lumican	Structural	22.3
	MIA3	Transport and Golgi organiaation protein 1 homolog	Secretory	15.8
	PAM	Peptidyl‐glycine alpha‐amidating monooxygenase	Enzyme	17.1
	PBXIP1	Pre‐B‐cell leukaemia transcription factor‐interacting protein 1	Transcription	17.2
	PON1	Serum paraoxonase/arylesterase 1	Enzyme	18.1
	SERPINF2	Alpha‐2‐antiplasmin	Protease	19.7
	SERPING1	Plasma protease C1 inhibitor	Adaptor	19.3
	TF	Serotransferrin	Transport	25.3
Fibroblast	CCDC80	Coiled‐coil domain‐containing protein 80	Extracellular matrix	15.8
	CEACAM1	Carcinoembryonic antigen‐related cell adhesion molecule 1	Cell adhesion	18.2
	CILP	Cartilage intermediate layer protein 1	Extracellular matrix	18.0
	COL3A1	Collagen alpha‐1(III) chain	Structural	17.7
	ECM1	Extracellular matrix protein 1	Extracellular matrix	18.0
	FGA	Fibrinogen alpha chain	Coagulation	24.2
	ITGA8	Integrin alpha‐8	Cell adhesion	17.6
	L1CAM	Neural cell adhesion molecule L1	Cell adhesion	18.1
	MATN2	Matrilin‐2	Extracellular	17.7
	PPIC	Peptidyl‐prolyl *cis*–*trans* isomerase C	Chaperone	19.2
	SERPINF1	Pigment epithelium‐derived factor	Protease	16.8
	TNXB	Tenascin XB	Structural	22.3
Cardiomyocyte and endothelial cell	ADAM9	Disintegrin and metalloproteinase domain‐containing protein 9	Metalloproteinase	17.5

*Note*: Proteins identified in the heart surfaceome and their comparison with cell (cardiomyocyte, fibroblast and endothelial cell) protein surface expression from the heart (PMID: 36950336).

Moreover, 225/701 proteins were reported as either or both cardiac cell surface proteins and heart tissue‐enriched proteins in Human Protein Atlas (HPA) [40, 41, 42] (Figure 3B, Table S12). A STRING‐based pathway enrichment analysis highlighted key networks associated with heart development (e.g., EGFR, DSG2, EPHB4, NRP1, NRP2, *p* value 2.5E‐22), cardiac conduction (e.g., PKP2, CAMK2D, CXADR, *p* value 1.7E‐05), cardiomyopathy (e.g., DAG1, CDH2, ITGB1, SGCD, PKP2, *p* value 1E‐13), EC proliferation (e.g., HSPG2, ADAM17, ECM1, *p* value 6.2E‐08) and wound healing (e.g., CD109, SERPING1, SERPIND1, SERPINC1, *p* value 2.7E‐15) (Table S12).

Further, 14/701 were validated as both cardiac cell surface proteins and heart tissue‐enriched proteins in HPA [40, 41] (Figure 3B, Table S12). Interestingly five proteins (BGN, CDH13, CDH2, ENG and FBLN2) were reported as EC surface [2] (Figure 3D). The protein expression of CDH2 in the heart is highly enriched relative to other tissues [43] (Figure 3E, Figure S3A, B). Further, using immunofluorescence, we analysed the cellular localisation of CDH2 and ENG to the plasma membrane surface and at cell junction from different human cell types [44] (Figure 3F). Thus, our data reports cardiac cell surface proteins accessible via circulation.

### 2.4 Heart‐Enriched Cell Surfaceome

**FIGURE 4:**
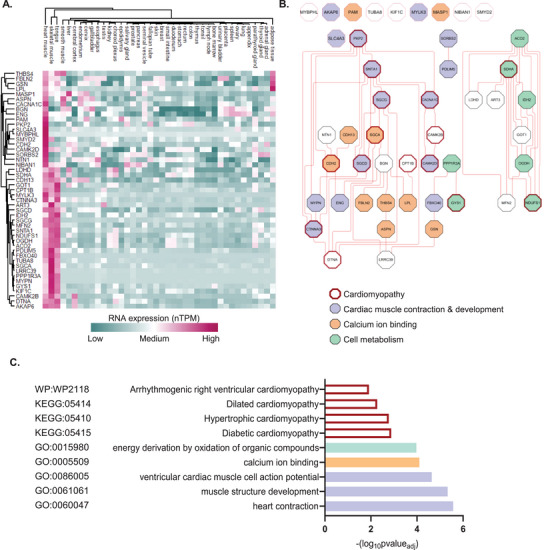
Heart surfaceome encompass heart‐enriched proteins. (A) Heatmap of heart surfaceome for their RNA expression (nTPM; transcripts per million) with protein evidence in organs elevated in the heart, based on data from the Human Protein Atlas [40]. (B) Protein–protein interaction network of the heart surfaceome; enriched cellular processes are highlighted. (C) Functional enrichment by Gene Ontology (GO) processes, KEGG and Wiki Pathways (significant, adj *p* value < 0.05) of heart surfaceome enriched in heart tissue.

We further dissected the 53 coronary accessible heart surfaceome proteins (Figure 3B), which were also enriched in heart tissue in HPA [40, 41, 45]. Indeed, our heatmap shows enrichment in the heart relative to other organs such as liver and small intestine, excretory organs (kidney, urinary bladder), reproductive organs, and brain and lung (Figure 4A, Table S14). We highlight 12 out of these 53 heart enriched surfaceome (Table 2), which includes CACNA1C, ENG, PAM, PKP2, SLC4A3, MYBPHL, SMYD2, CDH2, CAMK2D, SORBS2, NTN1 and NIBAN1.

**TABLE 2:** Heart‐enriched cell surfaceome.

Gene	Protein name	Protein function	Protein class
CACNA1C	Voltage‐dependent L‐type calcium channel subunit alpha‐1C (calcium channel)	Voltage‐gated calcium channel, cardiac muscle, contraction and signalling	Ion channel
ENG	Endoglin (cell surface MJ7/18 antigen)	Endothelial cell receptor, angiogenesis, vascular remodelling	Cell membrane receptor
PAM	Peptidyl‐glycine alpha‐amidating monooxygenase	Catalyses amidation of neuropeptides, neuroendocrine, protein synthesis	Enzyme
PKP2	Plakophilin‐2	Desmosome component, cell adhesion, cardiac tissue	Cell junction protein
SLC4A3	Anion exchange protein 3	Anion exchanger, pH regulation, bicarbonate transport	Ion transporter
MYBPHL	Myosin‐binding protein H‐like	Cardiac muscle, myosin binding protein, sarcomere organisation	Sarcomeric protein
SMYD2	N‐lysine methyltransferase SMYD2	Cardiomyocyte specific enzyme, Methylates histones, regulates transcription and heart muscle development	Histone methyltransferase
CDH2	Cadherin‐2 (neural cadherin) (N‐cadherin)	Calcium‐dependent, cell adhesion, cardiac and neural tissues	Cell adhesion molecule (calcium‐dependent)
CAMK2D	Calcium/calmodulin‐dependent protein kinase type II subunit beta (CaM kinase II subunit beta)	Calcium‐dependent kinase, heart signalling/contraction, regulatory protein signalling network	Cytosolic enzyme
SORBS2	Sorbin and SH3 domain containing 2	Scaffold protein, muscle cytoskeleton organisation, signalling	Scaffold protein
NTN1	Netrin‐1	Axon guidance, cell migration, cell survival	Extracellular signalling molecule
NIBAN1	Protein Niban 1 (protein FAM129A)	Cellular stress response, cell growth and homeostasis	Cytosolic protein

*Note*: Proteins identified in the heart surfaceome that were elevated in protein expression in heart tissue (nTPM) relative to other tissue expression (Human Protein Atlas, Tissue expression).

Importantly, GO enrichment analysis of these 53 proteins highlights cardiac cell signalling networks involving cardiomyopathy (CDH2, DTNA, PTKP2, SNTA1, CAM, K2D/B), cardiac muscle contraction and development (ENG, SNTA1, SGCG, MYPN), calcium ion binding (SGCA, MASP1, THBS4, FBLN2, GSN) and cell metabolism (SDHA, NUDFS1, GYS1, ACO2, IDH2) (Figure 4B, Table S14). These also include key plasma membrane structures such as ion channels and kinases (CACNA1C, SLC4A3, CAMK2D and PKP2), known to regulate cardiac muscle contraction and development [46, 47]. Other proteins essential in heart homeostasis include cell adhesion proteins like CDH2, ENG and SORBS, which mediate interactions with the ECM, facilitating calcium ion binding and cellular metabolism. These interactions are crucial for maintaining cell morphology, structural integrity and heart function. Additionally, proteins observed in the heart surfaceome are linked to cardiomyopathies (Figure 4C, Table S14), further highlighting the role of the heart cell surfaceome in cardiac health and disease.

## 3 Discussion

We have defined for the first time the cardiac cell surfaceome of the murine neo‐native (intact) heart. Proteins accessible to the vascular flow and associated with the heart tissue comprised a large number of adhesion molecules (including many classical endothelial markers), RTKs, enzymes, scavenger receptors, proteoglycans and plasma proteins. Although prior studies have demonstrated the use of systemic perfusion to tag murine vascular surfaces in vivo, using ester derivatives of biotin [27, 28, 38], these studies did not focus on the heart or blood/tissue interface through the coronary artery. Additionally, while studies have dissociated the heart and performed cell sorting to isolate and subsequently surface profile specific cell types from the heart [2, 25], such workflows disrupt the native anatomy of the heart and potential remodelling of cell surfaceome during isolation. The heart is comprised of numerous cell types, including CMs, FBs, SMCs and ECs, some of which are heart specific [48]. This study therefore provides a strategy to expand and decipher cardiac cell surfaceome that is accessible to the circulation.

To capture cell surface proteins, the vast majority of the chemical modification strategies utilise either a target approach (as *N*‐glycan moieties [2, 49]), an enzymatic shaving approach [50, 51] or workflows that modify the surface accessible side chains of various protein/peptide sequence residues (lysine or aspartic and glutamic acids) [52] on peripheral regions of the plasma membrane [30, 53]. Although selective in targeting a specific modification on the extracellular region, the target approach is limited based on the frequency of these sequences across all cell surface proteins and the requirement of starting cell material—restricting insights into global analyses of the cell surfaceome. We employed NHS‐SS‐Biotin as a thiol‐cleavable amine‐reactive biotinylation reagent as a flexible (broad pH, rapid incubation), global approach to capture the cell surface protein network [36].

Our workflow demonstrates a complex protein landscape embedded in the cardiac cell surfaceome. In our heart cell surfaceome (701 proteins, Table S10), we report adhesion, ECM and cytoskeletal network proteins including receptors (including RTKs), cell surface enzymes (e.g., proteases), bonified cell membrane cluster of differentiation components and various TM proteins including junctional molecules (cadherins, catenin), adhesion molecules (integrins), ephrins, plexins and sodium/calcium exchanger proteins. We further highlight cardiac cell‐enriched markers of principle heart cells and correlation with previous cell‐specific surfaceome studies of the cardiac cellular niche; cardiac FBs, CMs and coronary ECs [2]. We highlight that of the 108 proteins enriched in ECs, we identified 19/701 heart cell surfaceome that further associate with their annotation as vascular and cell surface, including classical endothelial markers VCAM1, INSR, CD34, PLVAP and CD109. Importantly, several of these proteins are linked with heart function and signalling cascades including GPI‐anchored protein CD109 antigen, JAM2, concentrated at cell‐to‐cell junctions in heart ECs of both large and small vessels, and VCAM1 (Table 3).

**TABLE 3:** Endothelial heart surfaceome.

Gene	Heart cell surfaceome	Endothelial cell surface N‐glycoproteins of cardiac cells, (Luecke et al. [2])	Protein name	Protein function
IGHM	Y	N/A	Immunoglobulin heavy constant mu	Immune response; endothelial interactions
AMBP	Y	N/A	Alpha‐1‐microglobulin/bikunin precursor	Reduces oxidative stress, inflammation
HRG	Y	N/A	Histidine‐rich glycoprotein	Regulates angiogenesis and remodelling
INSR*	Y	FB, CM, EC	Insulin receptor	Insulin signalling in endothelium.
ACE	Y	FB, CM, EC, SMC	Angiotensin‐converting enzyme	Regulates blood pressure, vasculature
TF	Y	N/A	Transferrin	Iron transport; affects angiogenesis
CD44	Y	FB, CM, EC, SMC	CD44 antigen (homing cell adhesion molecule)	Cell adhesion, migration, angiogenesis
VCAM1*	Y	FB, EC	Vascular cell adhesion molecule 1	Leucocyte adhesion during inflammation
ENPP1	Y	FB, EC, SMC	Ectonucleotide pyrophosphatase/phosphodiesterase 1	Regulates vascular calcification, barrier
CD34*	Y	FB, CM, EC, SMC	Hematopoietic progenitor cell antigen CD34	Endothelial progenitor cell marker
GPC1	Y	FB, CM, EC, SMC	Glypican‐1	Modulates endothelial growth signals
EPHA4*	Y	FB, CM, EC	Ephrin type‐A receptor 4	Endothelial adhesion, vascular development
JAM2*	Y	N/A	Junctional adhesion molecule B (JAM‐2)	Tight junction, barrier regulation.
EFNB1	Y	FB, EC, SMC	Ephrin‐B1	Vascular guidance, angiogenesis signalling
CD47*	Y	N/A	CD47 antigen (integrin‐associated protein)	Immune evasion, angiogenesis regulation
AOC3	Y	N/A	Amine oxidase copper‐containing 3	Vascular inflammation, leucocyte adhesion
CD109*	Y	FB, CM, EC, SMC	CD109 antigen	Regulates endothelial cell signalling
PXDN	Y	FB, EC, SMC	Peroxidasin homolog	Strengthens vascular basement membrane
PLVAP*	Y	FB, EC	Plasmalemma vesicle‐associated protein	Fenestrated endothelium; transport role

*Note*: Proteins identified in heart surfaceome, and heart endothelial cell sorted surface profiling, in addition to their annotation as vascular and cell surface (GO:009986). Protein (*)classical endothelial markers.

Abbreviations: CM, cardiomyocyte; FB, fibroblast; EC, endothelial cell; SMC, smooth muscle cell.

To understand cardiac‐specific functions of proteins linked to the heart and accessible from circulation, our data revealed 53 proteins commonly identified. These include 14 previously reported as cardiac cell surfaceome. These include two distinguished TM proteins, important in regulating intracellular calcium levels in cardiac cells. The CM voltage‐gated calcium channel, CACNA1C is crucial for maintaining cardiac muscle contractility and vascular blood flow [54]. Clinically, CACNA1C is a target for the treatment of hypertension [55] and arrhythmia [46]. The cell surface protein SLC3A2 (CD98hc), a member of the solute carrier family, is expressed on the surface of all cells in the heart and indirectly influences intracellular calcium levels through its role in amino acid transport [56]. Additionally, SLC3A2 is critical for vascular smooth muscle cell (VSMC) proliferation, and its deficiency has been linked to the formation of unstable plaques in atherosclerosis [57]. Interestingly, the conservation of both CACNA1C and SLC3A2 at the cardiac plasma membrane during cellular stress in the heart has not been fully elucidated [2].

Of note, ENG (CD105) has been prominently shown to localise to the endothelium of tumour‐associated vasculature. Additionally, CD105 has been identified as a targeting moiety for selective delivery to ECs in mouse blood vessels [58], and human CD105 promoter fragments have been used to drive CD59 expression in the small vessels of the heart, kidney and lung in pigs [59].

The heart is enriched with regulatory and structural proteins essential for coordinating molecular organisation and function. Our findings reveal 39 heart‐enriched proteins previously unrecognised as part of the cardiac cell surfaceome, many of which are critical for coronary vascular homeostasis. Among these, NTN1 and its receptors NEO1 and integrin α3β1 were identified, with NTN1 as a surfaceome marker enriched in heart tissue. Netrin‐1 has been shown in an in vivo study to inhibit leucocyte migration [60], suggesting in the heart, a cardioprotective role by reducing vascular inflammation after myocardial injury. Additionally, netrin‐1 promotes CM survival via interactions with vascular ECs [61], positioning these coronary surface proteins as potential therapeutic targets for cardioprotection.

Previously, in vivo phage display assay has been shown to identify peptide sequences that home to specific vascular sites in vivo [62]. Such approaches have been useful in generating tools for the targeted delivery of compounds to specific vascular ‘zip codes’, and they highlight the tremendous organotypic heterogeneity of the vascular surfaces. This current study provides a protein‐centric resource for cardiac cell surfaceome, and potential molecular leads for interaction and delivery to the murine neo‐native heart.

Similarly, 660 proteins from our heart surfaceome landscape have not been correlated with heart muscle tissue enriched. There are, however, 220/660 proteins listed as cardiac cell surfaceome [2]. For example, integrins (α1β1, α2β1), ephrins (EPHA4, EPHB4), cell‐junction proteins (ICAM1, VCAM1, JAM2) and a variety of disintegrin and metalloproteinase domains (ADAM10, ADAM17). Such proteomic signatures of the cardiac cell surfaceome have shared expression with the vasculature endothelium of other tissues [27, 28]. Therefore, these proteins may not be specific to the heart or cell types of the coronary vasculature surfaces. Since proteins in this study remain with unknown surface localisation or enrichment in heart tissue, further investigation of the heart cell surfaceome is warranted.

A non‐negligible fraction of identified proteins were intracellular proteins or serum components (from the heart surface, relative to the heart tissue). Sulfo‐NHS‐LC‐Biotin has been shown in in vitro studies to be capable of passing through cell membranes and to label intracellular proteins, in addition to surface proteins, but disulfide‐linked biotin derivatives such as Sulfo‐NHS‐SS‐Biotin yielded a more specific labelling of membrane proteins, because of disulfide bond cleavage in the reducing intracellular milieu [63]. In this study, we ensured minimal tissue diffusion of biotin and established co‐localisation of biotin with EC marker CD31. Optimising conditions associated with time, volume and chemical properties for perfusion and labelling may further influence which structures can be labelled and recovered in vivo. Importantly, by combining high‐resolution DIA‐based proteomics facilitated the identification of hundreds of different accessible proteins from the heart [64]. Importantly, such MS approaches are not biased towards abundant proteins/peptides, providing in‐depth targets for ligand‐based targeting applications, including low abundant tissue‐specific antigens, including endoglin (ENG, cell surface MJ7/18 antigen), CD166 antigen (ALCAM) and hematopoietic progenitor cell antigen CD34, in addition to antigens not previously associated with the cell surface HPA [39] including myeloid cell nuclear differentiation antigen‐like protein (MNDAL) and LIM and senescent cell antigen‐like‐containing domain protein (LIMS1).

Such antigens accessible from the vasculature are likely to be suitable targets for ligand‐based targeting applications, that may be perturbed in expression/localisation upon cardiac tissue remodelling associated with health, including aging [65] and disease [66, 67]. Indeed, LSMEM2 as a CM‐restricted cell surface protein has been highlighted negatively associated with human heart failure, and potential region‐specific marker for discriminating cellular phenotypes and disease states [2].

In conclusion, we have generated a global protein‐centric workflow that enables the labelling, capture and detection of the cardiac cell surfaceome, while retaining the neo‐native state of the heart. To the best of our knowledge, no prior studies have applied aortic cannulation combined with biotin labelling and proteomic profiling to attain the cardiac cell surfaceome. Data obtained using this versatile system highlights the proteomic labelling in the coronary vasculature, identification of cardiac‐specific proteomic signatures and heart‐enriched cell surface proteins with relevance to cardiac biology and function. This experimental workflow also provides a framework to understand remodelling of the cell surface of the heart and other vascularised organs and insights into mechanisms underlying physiological or pathological conditions of complex tissues including the heart.

## 4 Materials and Methods

### 4.1 Animal Studies

Animal experiments and access to hearts for perfusion were conducted in accordance with the guidelines and approval of the Alfred Research Alliance (ARA) Animal Ethics Committee, Vic, Australia (ethics approval number, P2580). Wild‐type male C57BL/6 mice were sourced from AMREP AS Pty Ltd, VIC. Eight mouse hearts representing biological replicates (divided into two independent analyses of *N* = 4 in each experiment set) were employed for mouse heart cell surfaceome analysis. A further validation round of independent mouse heart perfusions was performed (*N* = 3).

### 4.2 Murine Coronary Vasculature Perfusions (In Situ Biotinylation)

Briefly, animals were anaesthetised using isoflurane in a closed chamber, placed in the supine position and a median sternotomy was performed. A 25‐gauge blunted needle was used to cannulate the ascending aorta. A small cut was made in the inferior vena cava to allow drainage of perfusion solutions from the heart. All perfusion reagents were ice‐cold and infused using an infusion syringe pump; initially with PBS (2 mL, 0.5 mL/min) and biotin solution (1 mg/mL) containing EZ‐link Sulfo‐NHS‐SS‐Biotin of Pierce Cell Surface Biotinylation and Isolation Kit (A44390, Thermo Fisher Scientific) in PBS (2 mL, 1 mL/min). The last perfusion applied through the aortic cannula in the animals was a wash‐quenching solution (0.025 M Tris, 0.15 M NaCl; pH 7.2) at 2 mL/min. Control animals were perfused in the same way but with PBS.

### 4.3 Tissue Collection for Proteomic Analysis and Immunofluorescence Imaging

The mouse heart was immediately excised, the atria removed and the heart bathed in 30 mL PBS for 30 s before transversely sectioned into three parts. Two sections, designated for proteomic analysis, were placed into defined tubes and immediately snap‐frozen. The middle section, for immunofluorescence imaging, was coated with optimal cutting temperature (OCT) compound (Tissue‐Tek) and snap‐frozen as per the manufacturer's instruction. All tissue sections were stored at −80°C before analysis.

### 4.4 Heart Tissue Homogenisation and Lysis

Mouse sections allocated for proteomic analysis (∼100 mg) were combined with 1.1 mL of 3X homogenisation‐lysis buffer (50 mM Tris‐HCL, 2% SDS, 10 mM EDTA in PBS pH8) with HALT protease and phosphatase inhibitor (Thermo Fisher Scientific), as described [33] with the concentration adjusted for smaller working volume conditions. To each section, 3 × 3.2 mm stainless beads (precooled) were added, and tubes (on ice) were placed in the bead‐based homogeniser (Bullet Blender, 24 Gold, Next Advance, 120) and homogenised for 1 min (setting 8, 30 s pulse, placed on ice between each pulse). Additionally, the homogenate was probe sonicated using a micro tip (23 amp, 10 s) at 4°C. The resulting homogenates were centrifuged at 17,000 × *g* for 20 min to sediment insoluble tissue debris, with the supernatant quantified by microBCA protein assay (Thermo Scientific) as per manufacturer instructions and stored at −80 °C until further analysis.

### 4.5 Global Proteomic Sample Preparation

Global MS‐based proteomics of Global (Mock) and Global (Biotin) protein extracts (10 µg in 100 µL) (each with *N* = 4) was performed as previously described [68] using single‐pot solid‐phase‐enhanced sample preparation (SP3) method [69]. Briefly, samples were solubilised in 1% (v/v) sodium dodecyl sulphate (SDS), 50 mM HEPES pH 8.0, incubated at 95°C for 5 min and cooled. Samples were reduced with 10 mM DTT for 45 min at 25°C followed by alkylation with 20 mM indoleacetic acid (IAA) for 30 min at 25°C in the dark. The reaction was quenched to a final concentration of 20 mM DTT. Magnetic beads were prepared by mixing SpeedBeads magnetic carboxylate modified particles (65152105050250, 45152105050250, Cytiva) at 1:1 (v:v) ratio and washing twice with 200 µL MS‐water. Magnetic beads were reconstituted to a final concentration of 100 µg/µL and prepared as described [70].Protein‐bound magnetic beads were washed three times with 200 µL of 80% ethanol and reconstituted in 50 mM TEAB and digested with trypsin (Promega, V5111) at a 1:50 enzyme‐to‐substrate ratio for 16 h at 37°C with constant shaking (1000 rpm). The peptide mixture was acidified to a final concentration of 2% formic acid (pH ∼1–2) and centrifuged at 20,000 × *g* for 1 min. The peptide digests were frozen at −80°C and dried by vacuum centrifugation (Savant SPD121P, Thermo Fisher Scientific), reconstituted in 0.07% trifluoroacetic acid and quantified by Fluorometric Peptide Assay (23290, Thermo Fisher Scientific) as per manufacturer's instruction.

### 4.6 NeutrAvidin Protein Purification

The Biotinylated and Mock protein fractions were separated from their respective global proteomes following the manufacturer's recommendation for the Pierce Cell Surface Biotinylation and Isolation Kit (A44390, Thermo Fisher Scientific) as described [23] with minor changes. Ten micrograms of total protein extract (∼90 µL/sample) was incubated with NeutrAvidin (NA) resin (1:1; NA Agarose 50% slurry: sample) for 1 h at ambient temperature with end‐over‐end mixing on a rotor in a spin column lined with a 10 µm filter. Samples underwent four centrifuge spins at 1000 × *g* for 1 min. The first was the primary flow‐through, followed by three subsequent washes using the wash buffer of the Pierce Kit (A44390, Thermo Fisher Scientific) (∼100 µL/sample wash cycle). Biotin‐labelled proteins captured by the NA resin were released from the NA resin after a 1‐h incubation at ambient temperature and a further two‐step elution. First, 90 µL of 50 mM DTT in 1x HEPES was performed, followed by centrifugation for 2 min at 1000 × g, repeated with 20 µL and another round of centrifugation for 2 min at 1000 × *g*. The same elution steps were applied to the unlabelled biotin‐proteins (Mock) captured by the NA resin through non‐specific affinity binding [71].

Eluted proteins, linearised in 50 mM DTT, were alkylated with 110 µL of 50 mM IAA for 45 min at ambient temperature in the dark. The reaction was quenched to a final concentration of 50 mM DTT, before SP3‐based tryptic protein digestion (Biotin and Mock samples; each with *N* = 4) as per global proteome preparation as described [68, 69].

### 4.7 Liquid Chromatography and Data Independent Acquisition Mass Spectrometry

LC‐MS data acquisition was performed on Q Exactive HF‐X benchtop Orbitrap mass spectrometer coupled with UltiMate NCS‐3500RS nano‐HPLC and operated with Xcalibur software as previously described [70]. Peptides were bound to a trapping column (Acclaim PepMap100 C18 µm beads with 100 Å pore‐size, Thermo Fisher Scientific) in Buffer A (100% LC‐MS grade water, 0.1% FA) at 5 µL/min for 5 min in 55°C and separated by analytical column (1.9‐µm particle size C18, 0.075 × 250 mm, Nikkyo Technos Co. Ltd) with scheduled gradient of 2%–28% Buffer B (100% acetonitrile, 0.1% FA) for 40 min, 28%–80% for 2 min at 300 nL/min in 55°C (butterfly portfolio heater, Phoenix S&T). MS1 full scan was set to 60,000 resolution, 3e6 AGC target and maximum IT of 50 ms in 350–1100 *m*/*z* scan range. MS2 was set to 15,000 resolution, 1e6 AGC target and 27 ms maximum IT. A total of 38 scan windows with staggered 20 *m*/*z* isolation window from 350 to 1100 *m*/*z* with 28% normalised collision energy. MS‐based proteomics data is deposited to the ProteomeXchange Consortium via the MASSive partner repository and available via MASSive with an identifier (MSV000095912).

### 4.8 MS Data Processing and Analysis

DIA‐MS spectra were processed using DIA‐NN software [72] (v1.8) as previously reported [70, 73]. The DIA‐MS spectra were searched against the mouse proteome database (UP000000589, #55,319, June 2022) [37]. For library‐free searches, “FASTA digest for library‐free search/library generation” and “Deep learning‐based spectra, RTs and IMs prediction” were selected, and matching between runs (MBR) function was enabled. Trypsin/P was selected for enzymatic digestion with maximum 1 missed cleavage. The precursor change range was set to 1–4, and the *m*/*z* precursor range was set to 300–1800 for peptides consisting of 7–30 amino acids with N‐term methionine excision and cysteine carbamidomethylation enabled as a fixed modification and variable (no variable modification) modifications were kept as default. The mass spectra were analysed using default settings with an FDR of 1% for precursor identifications and match between runs (MBR) enabled for replicates.

Proteome data were further analysed using Perseus (v2.0.7.0) [74]. To ensure high quantitative confidence, proteins selected for downstream bioinformatics analysis include those that were identified in at least three out of four biological replicates (*N* = 4) in each experimental condition.

### 4.9 Bioinformatics and Statistics

Principal component analysis plot, volcano plot and protein rank plot were performed using Perseus. Bar charts were generated using GraphPad Prism (v10.0.2). GO‐based functional enrichment analysis (Biological, Molecular and Compartment pathways) was performed using g: Profiler [75], in addition to analyses using KEGG and Wiki Pathways. The Upset plot was generated using https://www.chiplot.online/. Selected protein interaction network visualised using Cytoscape software with STRING functional enrichment via StringApp [76]. Surfaceome (surface membrane) classification was performed using UniProt, previously experimentally verified CSPA [39] or predicted as surfaceome proteins based on SURFY [1]. Data analysed by a pair‐wise comparison test using Perseus and statistical significance defined using a Permutation‐based FDR (FDR < 0.05).

### 4.10 Immunofluorescence Staining and Microscopy

Preparation of fixed heart tissue sections for immunofluorescence staining and confocal imaging was performed by Monash Histology Platform, Clayton, Australia. The primary antibody included Goat pAb anti‐CD31 (1:300, R&D Systems, AF3628) and secondary antibody/labelled polymer Donkey anti‐Goat AF647 (Invitrogen, #A32849, 1:500) and Neutravidin (Thermo Fisher, #22832, 1:400). All slides were processed according to the service provider's specifications.

## Author Contributions

I.I. carried out the majority of experiments and data analysis. I.I. and A.R. metholodology and surfaceome enrichment strategy, D.D. performed biotin perfusions. I.I., A.R. and D.W.G. assisted with data processing, proteomic and surfaceome analyses, and interpretation. I.I., A.R. and D.W.G. wrote, reviewed and edited the manuscript. All authors approved the final manuscript.

## Conflicts of Interest

The authors declare no conflicts of interest.

## Supporting information



Supporting Information

Supporting Information

## Data Availability

The mass spectrometry proteomics data is deposited to the ProteomeXchange Consortium via the MASSive partner repository and available via MASSive with identifier (MSV000095912).
