# Supplementary material for: The Proteomic Landscape of the Coronary Accessible Heart Cell Surfaceome
Source: Proteomics. 2025 Jan 10;25(7):e202400320. doi: 10.1002/pmic.202400320 (PMC11962585; doi:10.1002/pmic.202400320)
Supplement: Supplementary file 1 — Supporting Information [file PMIC-25-e202400320-s001.pptx]

## Slide 1
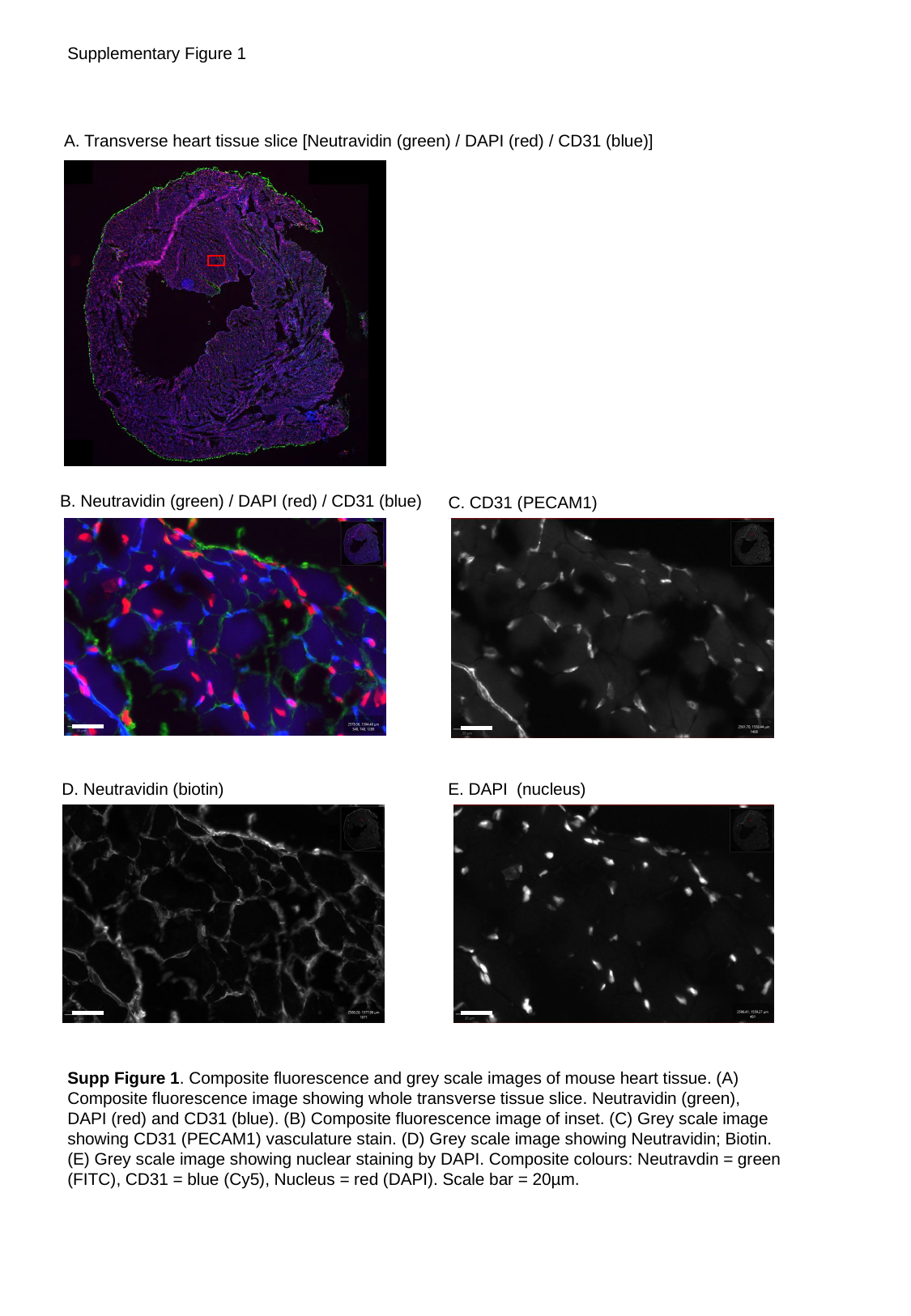

Supplementary Figure 1
A. Transverse heart tissue slice [Neutravidin (green) / DAPI (red) / CD31 (blue)]
B. Neutravidin (green) / DAPI (red) / CD31 (blue)
C. CD31 (PECAM1)
D. Neutravidin (biotin)
E. DAPI (nucleus)
Supp Figure 1. Composite fluorescence and grey scale images of mouse heart tissue. (A) Composite fluorescence image showing whole transverse tissue slice. Neutravidin (green), DAPI (red) and CD31 (blue). (B) Composite fluorescence image of inset. (C) Grey scale image showing CD31 (PECAM1) vasculature stain. (D) Grey scale image showing Neutravidin; Biotin. (E) Grey scale image showing nuclear staining by DAPI. Composite colours: Neutravdin = green (FITC), CD31 = blue (Cy5), Nucleus = red (DAPI). Scale bar = 20µm.

## Slide 2
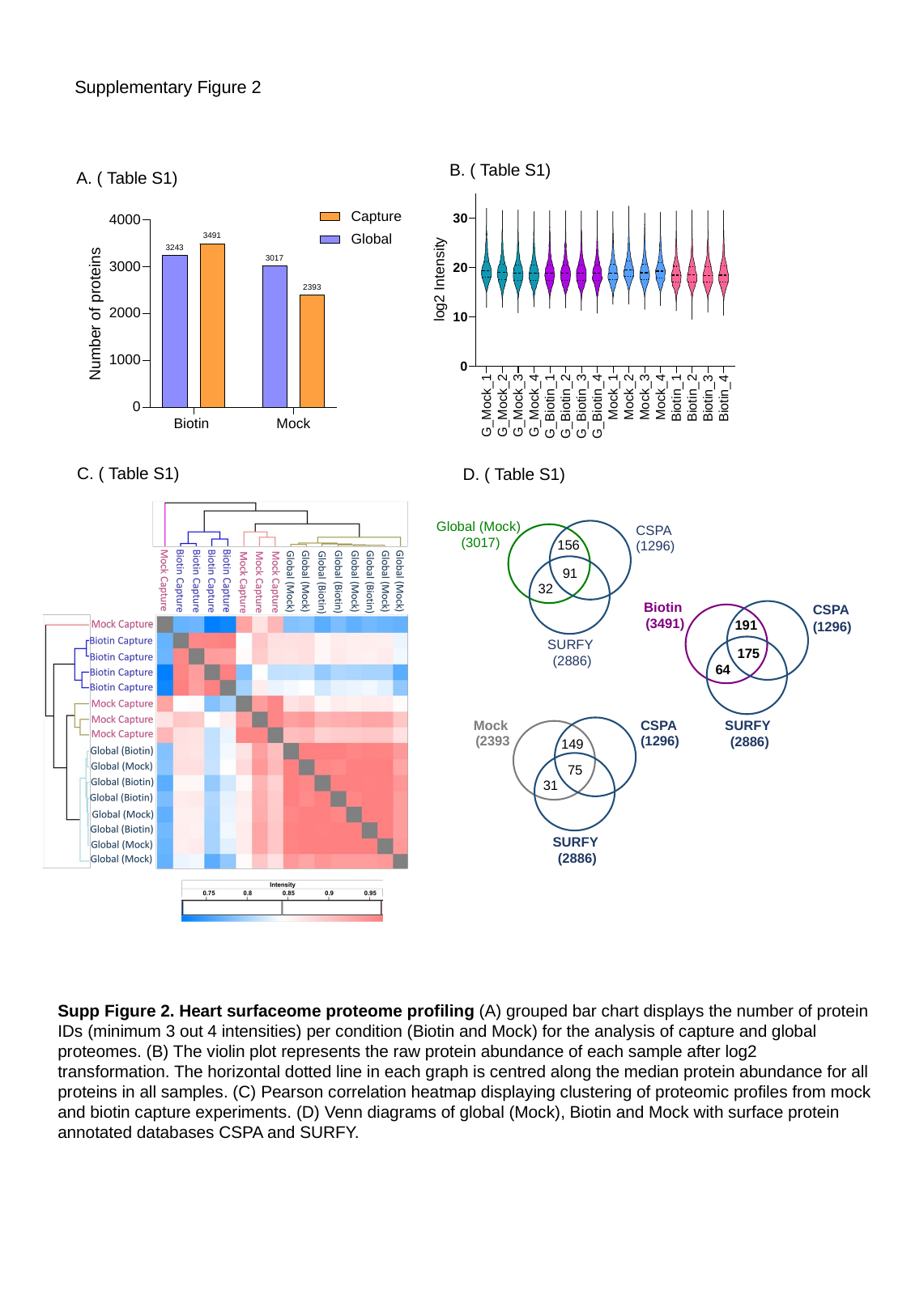

# Supplementary Figure 2
B. ( Table S1)
A. ( Table S1)
C. ( Table S1)
D. ( Table S1)
Global (Mock)
(3017)
CSPA
(1296)
156
91
32
SURFY
(2886)
Biotin
(3491)
CSPA
(1296)
191
175
64
SURFY
(2886)
Mock
(2393
CSPA
(1296)
149
75
31
SURFY
(2886)
Supp Figure 2. Heart surfaceome proteome profiling (A) grouped bar chart displays the number of protein IDs (minimum 3 out 4 intensities) per condition (Biotin and Mock) for the analysis of capture and global proteomes. (B) The violin plot represents the raw protein abundance of each sample after log2 transformation. The horizontal dotted line in each graph is centred along the median protein abundance for all proteins in all samples. (C) Pearson correlation heatmap displaying clustering of proteomic profiles from mock and biotin capture experiments. (D) Venn diagrams of global (Mock), Biotin and Mock with surface protein annotated databases CSPA and SURFY.

## Slide 3
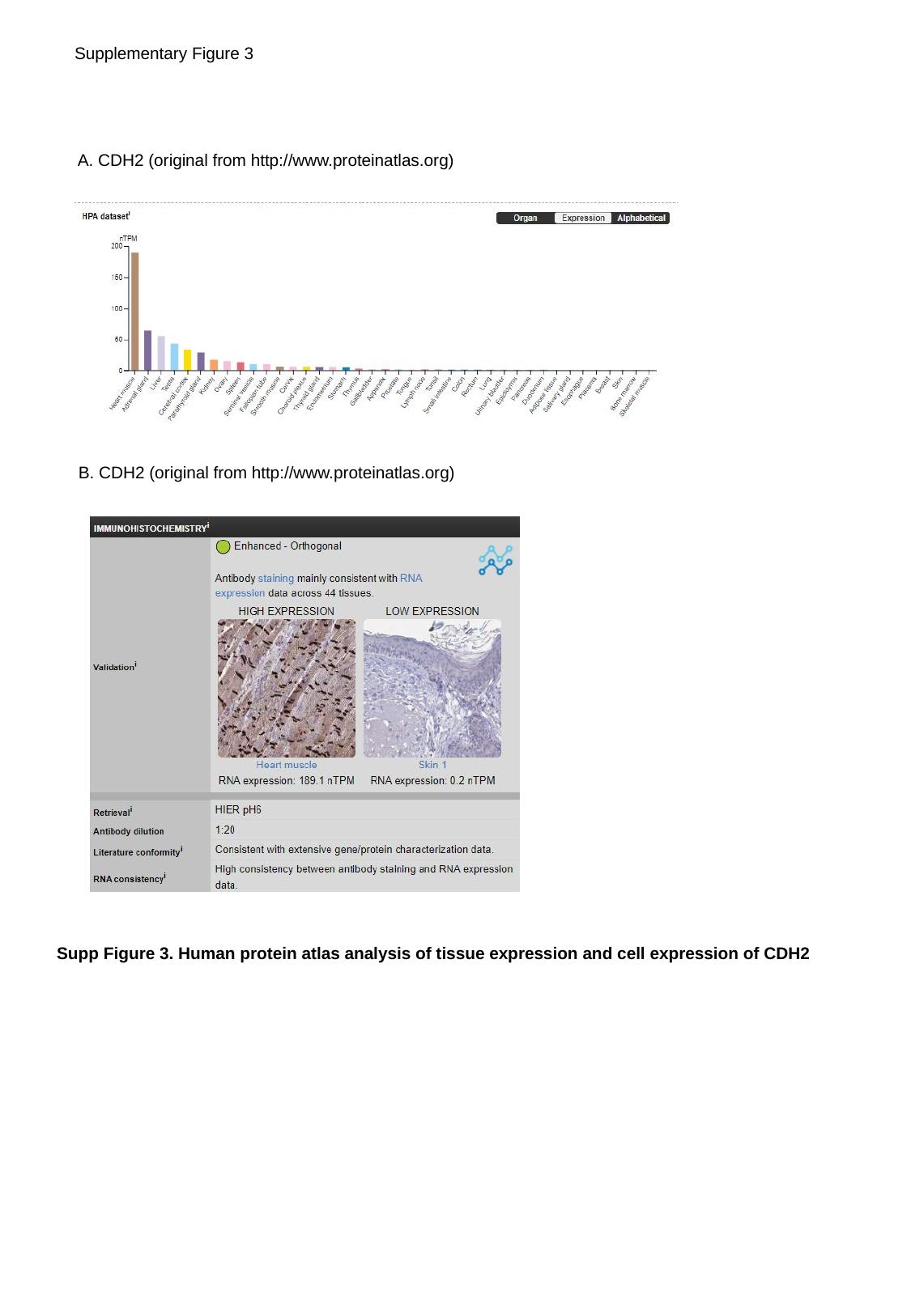

Supplementary Figure 3
A. CDH2 (original from http://www.proteinatlas.org)
B. CDH2 (original from http://www.proteinatlas.org)
Supp Figure 3. Human protein atlas analysis of tissue expression and cell expression of CDH2

## Slide 4
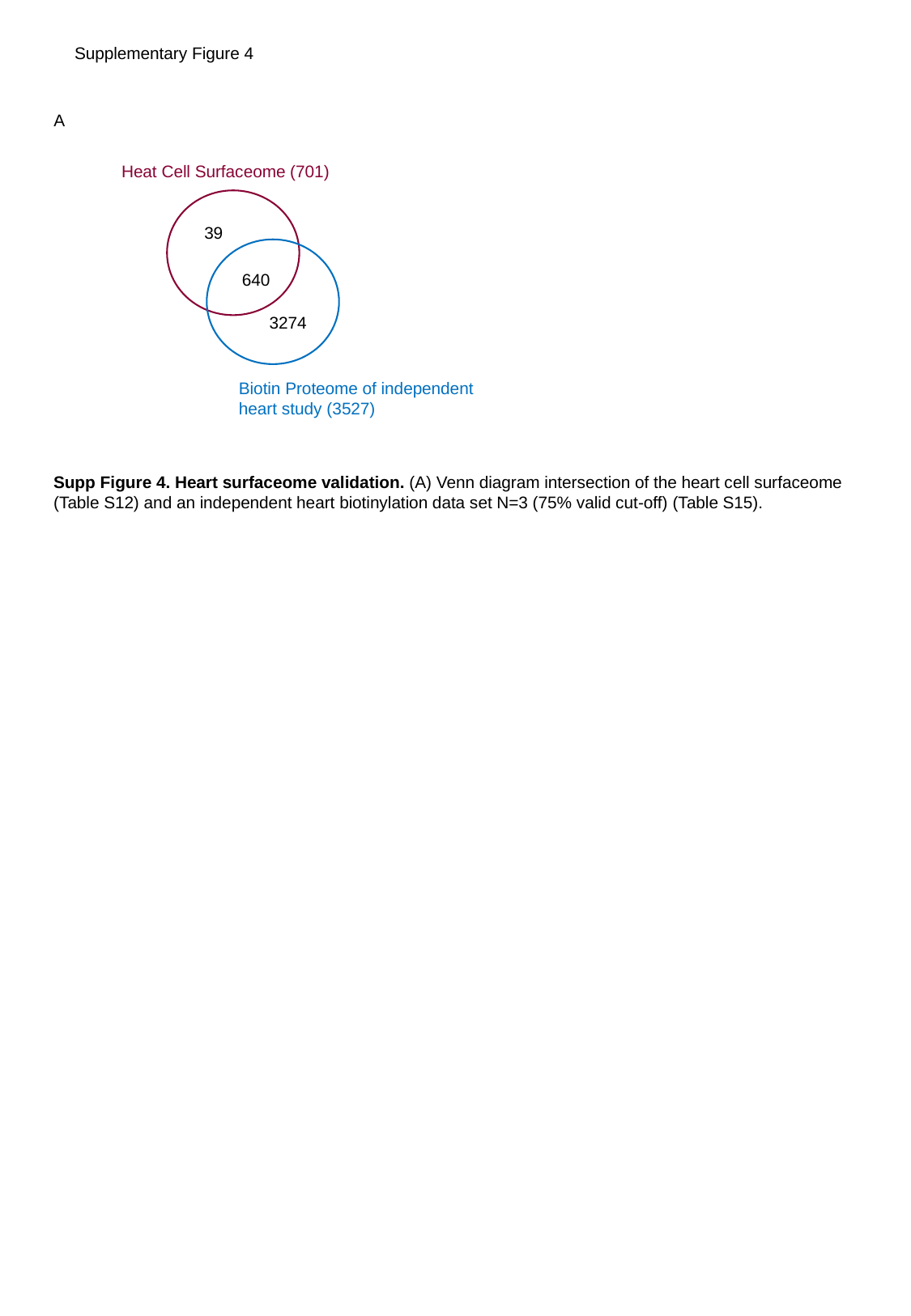

Supplementary Figure 4
A
Heat Cell Surfaceome (701)
39
640
3274
Biotin Proteome of independent
heart study (3527)
Supp Figure 4. Heart surfaceome validation. (A) Venn diagram intersection of the heart cell surfaceome (Table S12) and an independent heart biotinylation data set N=3 (75% valid cut-off) (Table S15).
